# Motivational Interviewing as a tool to enhance access to mental health treatment in adolescents with chronic medical conditions and need for psychological support (COACH-MI): study protocol for a clusterrandomised controlled trial

**DOI:** 10.1186/s13063-018-2997-5

**Published:** 2018-11-14

**Authors:** Christina Reinauer, Rabea Viermann, Katharina Förtsch, Hannah Linderskamp, Petra Warschburger, Reinhard W. Holl, Doris Staab, Kirsten Minden, Rainer Muche, Matthias Domhardt, Harald Baumeister, Thomas Meissner, COACH consortium

**Affiliations:** 1 Department of General Paediatrics, Neonatology and Paediatric Cardiology, University Children’s Hospital, Medical Faculty, Moorenstr. 5, Düsseldorf, 40225 Germany; 2 Department of Psychology, University of Potsdam, Potsdam, Germany; 3 Institute of Epidemiology and Medical Biometry, ZIBMT, University of Ulm, Ulm, Germany; 4 Division of Pulmonology and Immunology, Department of Paediatrics, Charité University Medicine Berlin, Campus Virchow Clinic, Berlin, Germany; 5 German Rheumatism Research Centre Berlin, Leibniz Institute, Berlin, Germany; 6 Department of Rheumatology and Clinical Immunology, Charité University Medicine Berlin, Berlin, Germany; 7 Institute of Epidemiology and Medical Biometry, University of Ulm, Ulm, Germany; 8 Clinical Psychology and Psychotherapy, University of Ulm, Ulm, Germany

**Keywords:** Adolescents, adherence to medical treatment, anxiety, chronic condition, depression, motivational interviewing

## Abstract

**Background:**

This cluster-randomised monocentric controlled trial focuses on improving the uptake symptoms of mental health care in adolescents with chronic medical conditions who have been identified by screening to have depression or anxiety. The study aims to determine the efficacy of motivational interviewing (MI) delivered by trained physicians to increase 12- to 20-year-old adolescents’ utilisation of psychological health care for symptoms of anxiety or depression.

**Methods/design:**

In this single-centre approach, *n* = 1,000 adolescents will be screened (using PHQ-9 and GAD-7), and adolescents with results indicative of anxiety or depressive symptoms (*n* = 162) will be advised to seek psychological health care in clusters from treating physicians in specialised outpatient departments. Participants who screen positive will receive either two sessions of MI or treatment as usual (TAU; regarded as the typical daily clinical practice), which is focused on recommending them to seek psychological health care for further evaluation. MI efficacy will be compared to the current TAU as the control condition. The primary outcome is the utilisation rate of psychological health care after counselling by an MI-trained physician vs. an untrained physician. Additionally, reasons for not claiming psychological support and changes in disease-related parameters will be evaluated in a 6-month follow-up session.

**Discussion:**

This trial will evaluate the feasibility of MI as a way to improve the utilisation of mental health-care services by adolescents who need further support other than that provided by standard care for chronic diseases. Physicians offering MI to adolescents may serve as a model for optimising health-care management in daily clinical practice, which may improve adolescents’ long-term well-being by improving adherence to medical treatment and preventing negative lifelong consequences into adulthood.

**Trial registration:**

German Trials Register (DRKS), DRKS00014043. Registered on 26 April 2018. Düsseldorf University study ID: 2017114504.

**Electronic supplementary material:**

The online version of this article (10.1186/s13063-018-2997-5) contains supplementary material, which is available to authorized users.

## Background

Adolescence is a challenging period with many health-related developmental tasks, risks and opportunities. Approximately 15% of German adolescents suffer from chronic medical conditions, such as asthma, diabetes and rheumatic diseases [1]. With these diseases, comorbid psychological symptoms, such as anxiety, depression and behavioural problems, are present in 10–40% of patients [2–6]. A complex interplay exists between anxiety and depression, disease attitude and treatment adherence. A screening measure for anxiety and depression has been introduced for some conditions, such as cystic fibrosis [2], but is not generally implemented for adolescents with other chronic diseases. In addition, no data exist on whether merely identifying anxiety or depression will result in improved mental health care. Furthermore, comorbid behavioural and emotional symptoms in adolescents with chronic conditions detrimentally affect medication adherence and adaption and increase the risk for negative long-term health outcomes [7]. Mental health issues are often neglected in current specialised medical care and physicians encounter substantial barriers in motivating adolescents to utilise psychological support [8]. Adolescents are highly resistant to considering psychological care for themselves [9]. Expert psychological interventions for coping with chronic conditions in the face of anxiety and depression have a robust evidence base [10, 11]; however, access to such services is only sporadic [12]. Referring adolescents to mental health care without appropriately motivating them often fails.

This study is part of the COACH trials consortium. COACH (Chronic Conditions in Adolescents: Implementation and Evaluation of Patient-centred Collaborative Health Care) is part of a nationwide research initiative to improve the mental health of children and adolescents, called *Gesund – ein Leben lang*, which is supported by the Federal Ministry of Education and Research, Germany. Consortium partners in Berlin, Potsdam, Ulm and Düsseldorf work together with different subprojects (such as motivational interviewing or MI in Düsseldorf). The steering committee is in Ulm. The aim of the COACH consortium is to demonstrate the efficacy of early behavioural interventions on health outcomes for adolescents with chronic medical conditions. First, the needs of adolescents with chronic medical conditions will be identified, and then the risk factors for comorbid mental health issues will be analysed. The long-term aim is to develop a model of collaborative care and pathways for disseminating and implementing early behavioural interventions into clinical practice.

The study on motivational interviewing (COACH-MI subproject) aims to determine the efficacy of an early intervention using MI for adolescents with chronic medical conditions to improve their utilisation of appropriate services, from paediatrics to mental health care, after early referrals for anxiety or depression.

MI is a collaborative evidence-based counselling technique designed to intrinsically motivate and strengthen patients’ commitment to improving a range of health behaviours [13–15]. MI has previously been shown to be effective in adolescent populations. It has been evaluated across different chronic medical conditions and positively affects the uptake of cognitive behavioural therapy (CBT) [16–22]. In the latter study [22], a clinical psychologist with postgraduate MI training conducted individual MI and active control (befriending) sessions with adolescents who had been diagnosed as having mood or anxiety disorders to improve treatment engagement in a standard therapy setting (group CBT). The new aspect of our study is that MI will be delivered by physicians treating patients with chronic conditions who have screened positive for anxiety and depression symptoms to decrease these adolescents’ unwillingness and concerns regarding psychological health care.

If the intervention is superior to treatment as usual (TAU), MI may be taught to and implemented by physicians in paediatric care, at least for those physicians who treat patients with chronic conditions. In the long term, stabilising mental conditions in adolescents through an early referral to mental health care could lead to better treatment adherence and self-management in chronic conditions, thus preventing long-term health consequences. Currently, physicians are often untrained in patient-centred communication with adolescents.

### Objectives

This study will determine the efficacy of providing adolescents with MI sessions administered by MI-trained physicians to improve mental health-care uptake rates. Patients who screen positive for anxiety or depressive symptoms will have two MI sessions with a trained physician or TAU (by a physician untrained in MI). We expect adolescents to benefit from conversations utilising the MI approach. The primary objective is to test whether MI increases the utilisation of supportive psychological counselling in patients with symptoms of anxiety or depression. For those individuals who do not seek psychological health care, we will analyse the reasons why the recommendations were not followed. Secondary outcomes, which will be measured at the 6-month follow-up, include improved symptoms of anxiety and depression and treatment-related parameters. Furthermore, treatment fidelity and the success of brief MI training sessions for physicians in our facility will be analysed by evaluating the interviews. The prevalence of anxiety and depressive symptoms will be analysed for a broad spectrum of chronic diseases.

### Study design

**Fig. 1:**
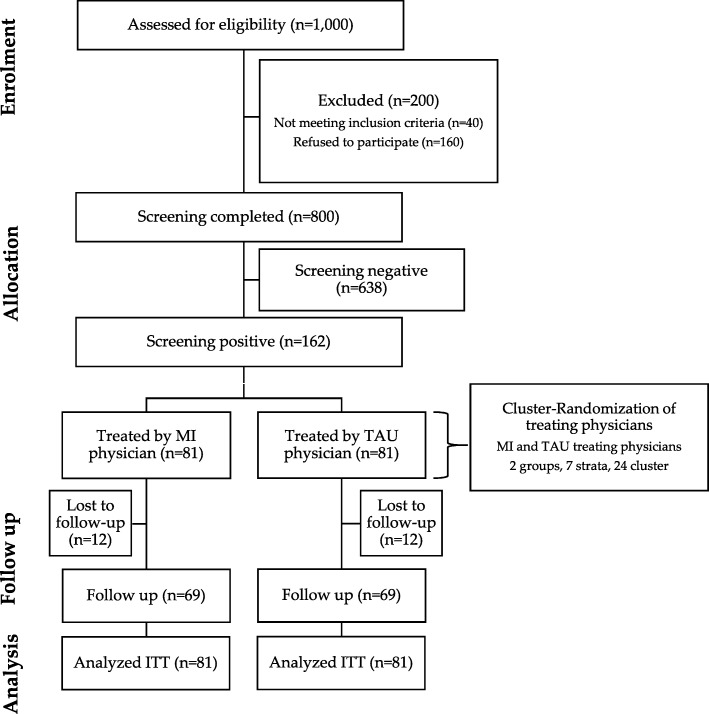
Flow chart of study design. ITT intention to treat, MI motivational interviewing, TAU treatment as usual

This study will be conducted as a pragmatic cluster-randomised monocentric controlled trial at the University Children’s Hospital Düsseldorf with two parallel groups (Fig. 1).

## Methods/design

### Eligibility criteria

#### Inclusion criteria

Adolescents with chronic medical conditions will be eligible for mental health screening if they are between 12 and 20 years of age. Chronic medical conditions are diseases that persist for >1 year. They significantly impair the patient’s daily routine and require continuous care and/or medical treatment.

To achieve high external validity, we plan to screen all patients attending the outpatient department of the University Children’s Hospital, Düsseldorf, Germany, who fulfil the inclusion criteria, and we will randomise all physicians who oversee the medical care of these patients.

Patients who screen positive with depression or anxiety symptoms will be counselled by their treating physician, who will have previously been randomised to perform either MI or TAU.

#### Exclusion criteria

Participants will be excluded if they are currently attending regular psychotherapy at the time of recruitment, or those with psychosis, acute suicidality, severe intellectual disability (IQ < 70), inability to communicate (verbally or in writing) or who currently abuse alcohol or drugs.

### Study interventions

#### Screening

Anxiety and depression will be screened as part of the clinical routine. After providing informed consent, participants will complete the following screening questionnaires on a tablet computer in the outpatient clinic immediately prior to their scheduled appointment: the Generalised Anxiety Disorder 7-item scale (GAD-7) [23] and the depression portion of the Patient Health Questionnaire (PHQ-9) [24–26]. Patient-reported outcomes on disease-related parameters will be assessed after the standardised screening questionnaires (see below).

Immediate feedback will be provided based on the results of these screening tools. Positive screening, defined as either a GAD-7 or PHQ-9 score ≥ 7, will trigger a recommendation for supportive counselling (see Section 1.2). Negative screening results will be conveyed to the adolescent and a psychoeducational handout will be provided.

#### Training of the treating physicians in motivational interviewing

As a prerequisite, physicians randomised to the MI group will attend a 2-day MI training course certified by the Motivational Interviewing Network of Trainers. Their ability to practise MI will be evaluated based on recorded counselling sessions in a standard procedure known as the motivational interviewing treatment integrity check. The results will be delivered to and discussed with the physician to improve their MI skills. A 2-day course in MI has been shown to provide sufficient and successful training to clinicians [27].

Patients being treated by MI physicians who screen positive will be provided with two counselling sessions implementing MI. The first MI session will be conducted immediately after the screening and will last 15–50 min. Sessions will be audio-recorded after mutual agreement. A second appointment will be scheduled within 2–4 weeks for a second 30–50 min MI session. MI aims to engage the patient in discussing the option for professional psychological support, focusing on their thoughts when considering this option and discussing possible barriers as well as pros and cons. This technique will be used to consider change as an option and to change their behaviour, i.e. to meet with a psychotherapist or other health-care provider delivering psychological treatment or psychotherapy in our facility.

#### Treatment as usual

The control condition is TAU, which includes providing immediate advice after the screening to seek psychological support. The physician will inform the patient of the assumed need for mental health care based on the screening questionnaire results, without specifically considering the adolescent’s perspective or potential barriers to the adolescent using this recommended support. The TAU physicians will perform their usual standard of care. They are free to deliver their counselling with no requirements regarding time span, content or conversation techniques. The duration and content of the conversation after a positive screening result will be briefly documented by the physicians.

The study physicians in both groups will provide a standardised written recommendation for the patient to seek specialised mental health counselling, specifically by psychotherapists. The document contains the addresses of local offices that can schedule psychological or mental health care appointments.

Both MI and TAU conversations will be audio-recorded for qualitative and quantitative analyses by mutual consent.

### Outcomes

#### Primary outcome

**Fig. 2:**
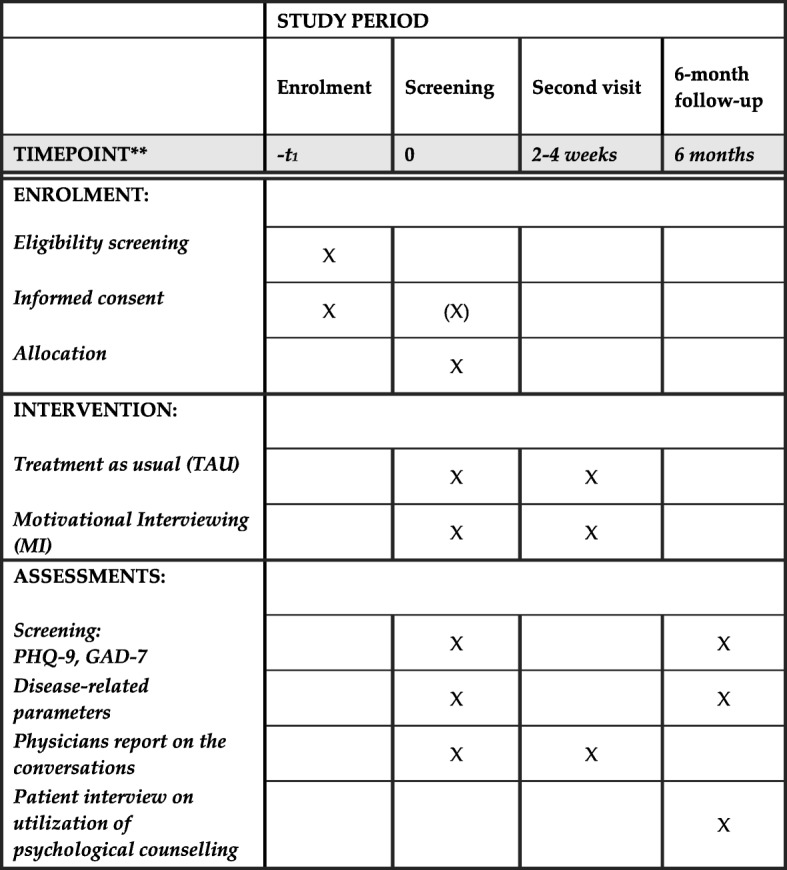
SPIRIT schedule of enrolment, intervention and assessments

The primary outcome is the utilisation of psychological health care. All patients with positive screening results are recommended to seek psychological counselling or psychotherapy. Successful referral is any appointment for psychological health care, defined as counselling with a (child and adolescent) psychotherapist, psychologist, psychiatrist or internet-based CBT counsellor in at least one face-to-face or digitally delivered session of psychological treatment within the 6-month follow-up interval. Patients on waiting lists (for psychological health-care appointments) will be counted as a positive outcome and will be reported separately. The primary outcome will be assessed via a semi-structured interview (telephone or face-to-face during a follow-up visit, Fig. 2) by blinded independent assessors unaware of the patient’s allocation.

#### Secondary outcomes

The secondary outcomes include baseline changes in anxiety and depression scores (GAD-7 and PHQ-9), disease-related parameters and the number of sessions attended by the follow-up. The follow-up interview will gather concrete data on the counsellor thereby enabling us to evaluate which form of psychological support (psychotherapy, counselling, psychiatric treatment or internet-based CBT) was used or to identify patients’ reasons for not using psychological health care.

Besides anthropometric data (age, gender, height, weight, body mass index and disease duration), the following disease-related parameters will be assessed: the need for technical aids (e.g. a wheelchair or oxygen), daily treatment duration, global assessments of limitations in daily life and pain, days absent from school or work, missed clinical visits and adherence measures using the Medication Adherence Rating Scale, German version (MARS-D) [28]. For diabetes (HbA1c), lung diseases (FEV1 expected) and juvenile idiopathic arthritis (Juvenile Arthritis Disease Activity Score) disease-specific parameters will be assessed. We will examine possible serious adverse events (SAEs) associated with the present screening and MI education approach. Gender-specific results will be analysed.

Audio-recorded MI/TAU conversations will also be quantitatively and qualitatively analysed using standardised tools (e.g. the motivational interviewing treatment integrity check) to assess treatment fidelity.

#### Screening measures

##### Generalised Anxiety Disorder Screener

GAD-7 is a practical self-report anxiety questionnaire. The occurrence of the seven core symptoms of generalised anxiety disorder (DSM-5) can be scored from 0 = ‘not at all’ to 3 = ‘nearly every day’ during the last 2 weeks. GAD-7 scores range from 0 to 21. Good internal consistency is reported with Cronbach’s α between .79 and .91 [29]. GAD-7 has been successfully used in adolescents [30, 31]. For the current study, a cut-off of ≥7 will qualify as a positive result [32–34]. A threshold of 15 points will indicate a red flag for severe anxiety symptoms [35].

##### Patient Health Questionnaire

In the PHQ-9 depression module, which is derived from the full Patient Health Questionnaire, nine DSM-5 criteria are scored from 0 = ‘not at all’ to 3 = ‘nearly every day’. Internal reliability estimates range from .86 to .89 using Cronbach’s α, and the 2-day test–retest reliability is estimated at .84 with nearly identical mean total scores [36]. For the current study, a cut-off of ≥7 will qualify for a recommendation of psychological treatment, as a score of >7 includes patients with major or minor depression [26, 37]. A cut-off of 20 points is the threshold for a red flag for major depressive symptoms [36].

#### Study design

##### Randomisation

Prior to enrolling the first participant, treating physicians in the specialised outpatient departments (Paediatric Allergy/Pulmonology, Diabetes/Endocrinology, Metabolic Diseases, Paediatric Cardiology, Gastroenterology, Rheumatology/Immunology and Paediatric Neurology) will be cluster-randomised to perform either MI or TAU. The dynamic allocation method of Pocock and Simon [38] will be used for randomisation and to estimate recruitment rates per physician (high or low) among the departments. The randomisation will be performed by an independent institute and blinded for the involved physicians [39]. MI-randomised physicians will sign a confidentiality agreement regarding MI to prevent potential trial-arm contamination.

##### Trial intervention and allocation

Patients will be recruited over 24 months. Eligible adolescents with chronic disease will be enrolled at the Düsseldorf University Children’s Hospital outpatient clinic. Patients and caregivers will be informed of the study prior to the clinical appointment.

The screening results will be displayed on the tablet computer to the treating physician with colour indicators (red flag: suicidality, major depression or severe anxiety, yellow: positive, green: negative), and the physician will discuss the result with the patient and their caregivers. Those with negative screening results will be briefly advised that psychological care is currently unnecessary for anxiety and depression, and a psychoeducational handout on coping with chronic conditions will be provided.

All participants with positive screening results (GAD or PHQ ≥ 7) will receive standardised written feedback with a recommendation to seek psychological counselling, They will be given contact addresses for local medical appointment schedulers. These participants will be allocated to either an MI or TAU intervention, depending on their treating physician’s randomisation. MI and TAU will be performed within the appointment framework. All patients will return for a second appointment within 2–4 weeks after inclusion (Fig. 2).

##### Follow-up

Six months after study enrolment and their first MI or TAU session, the patients will be interviewed regarding their psychological counselling (see primary outcomes). Utilisation of psychological health care will be recorded in detail (number and type of sessions and missed appointments). Secondary outcomes will be assessed in the interview and by repeating the initial questionnaires (tablet, online or paper and pencil). Clinical data will be obtained from the most recent clinical visit.

#### Sample size

The proposed sample size for the baseline mental health screening assessment in our monocentric study is estimated at approximately *n* = 1,000 cases in 24 months. The prevalence of depression or anxiety symptoms is estimated at 15–20% in total across the disease spectrum [2] (Fig. 1).

The rate of successful mental health-care referrals for TAU is estimated at 10% [40] and we expect an increase to at least 30% for the intervention group. For a two-sided chi-square test with a power of 80% and a significance level of 5%, the software NQuery 8.0 (Statistical Solutions, 2018, Cork, Ireland) gives a sample size of *n* = 62 per group. We will correct the sample size by 10% for cluster effects, resulting in an estimated intra-cluster correlation coefficient of 2.5% and a sample size of *n* = 69 per group. To adjust for a 15% drop-out rate, the sample size per group is targeted at *n* = 81, treated by either MI physicians or TAU physicians, for a total of *n* = 162 patients (Fig. 1). All cases included in the study will be analysed by an intention-to-treat analysis.

### Data collection methods

Data on depression, anxiety (PHQ-9 and GAD-7), days absent from school or work, daily treatment duration, global assessments of limitations in daily life and pain, and adherence (MARS-D) will be collected directly from the patients (patient-reported outcomes) by a tablet questionnaire. Pseudonymised results will be stored on a secure local and national server. Physicians responsible for treating the participants medically will contribute additional medical information on case report forms at baseline and at the 6-month follow-up.

### Data management and monitoring

#### Data analysis

The primary outcome will be confirmed by a logistic mixed model adjusting for the cluster structure in the data at a two-sided significance level of 5% [41]. Furthermore, the main outcome will undergo an exploratory analysis using logistic regression to adjust for covariates, such as age, sex and medical condition. The analyses will be performed in the intention-to-treat population.

Secondary measures will be analysed using non-parametric tests (number of psychological face-to-face or online sessions attended within the 6-month follow-up interval, missed clinical visits, acceptance to participate in the study and gender) and mixed ANOVAs (disease-related parameters and GAD-7, PHQ-9 and MARS-D scores).

Treatment safety will be analysed by comparing the SAE rates between groups by Fisher’s exact test.

### Ethics and dissemination

#### Dissemination plan and data management

Pseudonymised study data will be stored on a secure local server and transferred to the central data server in Ulm, Germany, using appropriate safety measures. Data safety will be ensured according to German data protection regulations. Study results will be presented at national and international conferences and will be published in peer-reviewed journals.

If the intervention is found to be superior to TAU, physicians may be educated in MI, and MI may be implemented in paediatric care, at least for those physicians who treat patients with chronic conditions.

Representatives from different patient organisations (young patients and caregivers) will be invited to participate in the consortium’s stakeholder advisory board. The advisory board will play an important role by providing input during the drafting of the translational reports by the research group for the funding agency and for the public.

## Discussion

This study design allows us to assess the efficacy of physicians’ MI education and spending more time on counselling in comparison to TAU when referring patients from paediatrics to mental health care. Because the MI approach appreciates and respects adolescents’ personal characteristics, motivations and perspectives, it is well suited for use during transition periods, such as adolescence and young adulthood, during which autonomy is an important developmental task.

While the European Cystic Fibrosis Society recently added mental health screening to its guidelines, this screening is not yet standard for other chronic diseases of adolescents in Germany. Currently, physicians are often untrained in patient-centred communication with adolescents. In the long-term view, stabilising mental conditions in adolescents through an early referral to mental health care could lead to better control of chronic conditions and the prevention of lifelong health consequences.

Early referral and motivation to receive psychological counselling for anxiety or depression is presumed to reduce these comorbidities. In addition, it may improve long-term adherence to therapy and may change maladaptive dysfunctional health behaviours, which can become chronic at this age. One goal is to prevent negative long-term health consequences. We will analyse methods to overcome individual and structural barriers to the uptake of mental health services, which should be identified and reduced in the long term.

A major limitation of our study is that due to the organisation of daily clinical practice, comparing MI with TAU is possible only by providing additional time for the MI trial arm. MI physicians will schedule a second appointment. In contrast, providing second visits for patients treated by TAU physicians is not the current standard of care. Eventually, if MI proves effective, we will be unable to differentiate between (i) a pure MI effect and (ii) the additional effect of treatment time or the increased attentiveness and warmth of the physician. This should be addressed in future studies. A further limitation of our study is that our screening does not record patients who may need mental health care for reasons other than anxiety or depression.

If the intervention evaluated in this study is effective, routine screening for anxiety or depression as well as routinely educating physicians in MI may be implemented in routine clinical care to reduce barriers and increase the utilisation of mental health services.

## Additional file

Additional file 1: SPIRIT 2013 Checklist: recommended items to address in a clinical trial protocol and related documents. (DOC 122 kb)

## Abbreviations

CBT: Cognitive behavioural therapy
COACH: Chronic Conditions in Adolescents: Implementation and Evaluation of Patient-centred Collaborative Health Care
GAD: Generalised Anxiety Disorder Screener
MARS-D: Medication Adherence Rating Scale, German version
MI: Motivational interviewing
PHQ: Patient health questionnaire
SAE: Serious adverse event
TAU: Treatment as usual
